# Activation of TLR4 signaling promotes gastric cancer progression by inducing mitochondrial ROS production

**DOI:** 10.1038/cddis.2013.334

**Published:** 2013-09-12

**Authors:** X Yuan, Y Zhou, W Wang, J Li, G Xie, Y Zhao, D Xu, L Shen

**Affiliations:** 1 Department of Clinical Laboratory, Xinhua Hospital, Shanghai Jiaotong University School of Medicine, Shanghai 200092, China; 2 Monash Institute of Medical Research, Monash University, Clayton, Victoria 3168, Australia; 3 Institute of Ageing Research, Hangzhou Normal University School of Medicine, Hangzhou 310036, China

**Keywords:** TLR4, gastric cancer, mitochondrial ROS, progression

## Abstract

Chronic infection, such as *Helicobacter pylori* infection, has been associated with the development of gastric cancer (GC). Pathogen-associated molecular patterns can trigger inflammatory responses via Toll-like receptors (TLRs) in GC. Here we showed that Toll-like receptor 4 (TLR4) was highly expressed in GC cells and was associated with the aggressiveness of GC. The binding of lipopolysaccharide (LPS) to TLR4 on GC cells enhanced proliferation without affecting apoptosis. Higher level of reactive oxygen species (ROS) was induced after activation of TLR4 signaling in GC. Using oxidase inhibitors and antioxidants, we found that mitochondrial ROS (mROS) was major source of TLR4-stimulated ROS generation. This elevated mROS production can be inhibited by diphenylene iodonium (DPI), and the blocking of the mROS production rather than ROS neutralization resulted in cell cycle arrest and the loss of mitochondrial potential, which were plausible reason for decreased cell viability. Furthermore, the increased mROS owing to TLR4 signaling resulted in the activation of Akt phosphorylation and NF-*κ*B p65 nuclear translocation. Altogether, these results reveal a novel pathway linking innate immune signaling to GC cell proliferation, implicate mROS as an important component of cell survival signals and further establish mitochondria as hubs for GC therapies.

Although the incidence of gastric cancer (GC) has decreased over the last decades, it is still one of the most frequently occurring digestive tract cancers and has a poor prognosis and a high mortality rate worldwide, especially in Asian countries.^1, 2^ Therefore, understanding the detailed mechanism of the development and progression of GC would be helpful to improve treatment. Epidemiological studies suggest that chronic inflammation has a significant role in the development of GC.^3^

There are a large number of studies demonstrating a key role for Toll-like receptors (TLRs) and innate immune responses in inflammation-associated carcinogenesis. Because TLRs have a pivotal role in immune responses to pathogens, most previous research on TLRs biology focused on immune cells.^4^ In addition to immune cells, some studies explored the function and biological importance of TLRs expressed in tumor cells. Recent reports indicated that genetic variations between TLR2, TLR3 and Toll-like receptor 4 (TLR4) were associated with colon and rectal cancer risk.^5^ TLR4 signaling promoted tumor growth in ovarian cancer.^6^ TLR2 was shown to be involved in the oncogenic function of STAT3 in gastric carcinogenesis.^7^ TLR4 was required for the promotion of hepatocellular carcinoma.^8^ The triggering of TLR4 and TLR9 in prostate cancer cells has also been shown to contribute to the malignant transformation of benign prostate epithelia.^9^ Chronic infection with *H. pylori* increases TLR4 expression in gastric epithelial cells, and TLR4 signaling in GC cells may be associated with the subversion of host defense mechanisms and the progression of cancer.^10^ GC cell express TLR4, which augments nuclear factor-*κ*B (NF-*κ*B) activation upon recognition of *H. pylori* lipopolysaccharide (LPS).^11, 12^ Although the expression of TLR4 in GC cells has been examined, the detailed mechanisms and the molecular pathways mediated by TLR4 signaling in gastric tumorigenesis are still not fully elucidated.

There is considerable evidence suggesting that reactive oxygen species (ROS) are essential components of the innate immune response against intracellular bacteria and that oxidative stress is associated with several pathological conditions, including chronic inflammation, infection and cancer.^13, 14^ Recent research has shown that the engagement of TLRs augments ROS production and enhances macrophage activity.^15^ Compared with normal cells, cancer cells have increased metabolisms and generate more ROS, which affect cell survival. Several studies have suggested that ROS can act as secondary messengers and control various signaling cascades, leading to sustained proliferation of cancer cells. Increased ROS generation accounted for the malignant phenotype of the cancer cells.^16^ As for GC, there was evidence that *H. pylori*-infected gastric epithelial cells generated ROS, which have an important role in gastric carcinogenesis.^17^ There is growing interest in ROS signaling in gastric carcinogenesis; however, the mechanisms and the pathways responsible for ROS production in GC remain unknown.

In this study, we investigated the effect of TLR4 signaling on the growth of GC cells. We evaluated ROS level and the mechanism associated with ROS generation after TLR4 signaling and GC cell proliferation. Oxidase inhibitors and antioxidants were used to explain the source of TLR4-stimulated ROS generation and evaluate the effect of ROS on the proliferation of GC cells. Finally, we explored the underlying molecular mechanism involved in mitochondrial ROS (mROS) increase via TLR4 signaling. This work deepens our understanding of the association between innate immune signaling and GC cell proliferation network and offers new strategy for GC therapy.

## Results

### TLR4 are expressed by GC cells and correlate with tumor stage

**Figure 1:**
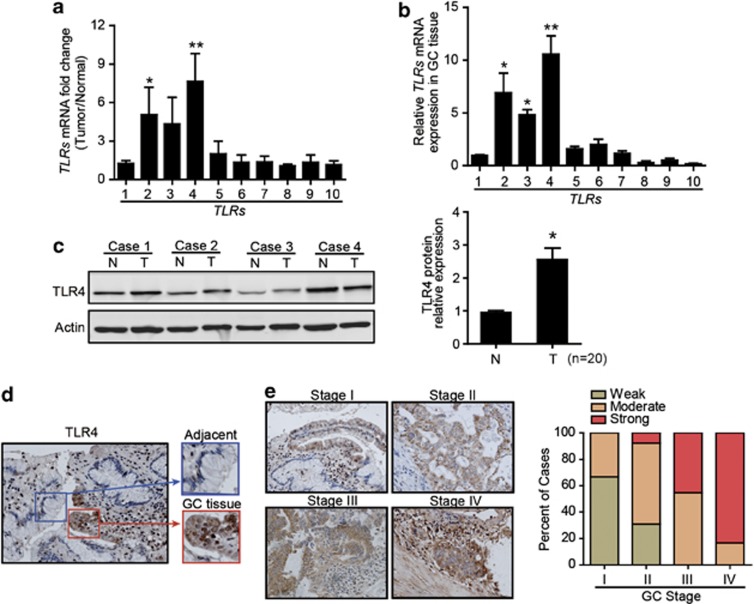
The elevated expression of TLR4 in GC tissues. (**a**) The ratios of mRNA levels for a panel of *TLRs* in GC tissues *versus* axillary normal gastric tissues were shown (*n*=10) (*P<0.05; **P<0.01, compared with TLR1). (**b**) Relative expression of *TLRs* (*TLR1-10*) mRNA in GC tissues were shown (*n*=10) (*P<0.05; **P<0.01). (**c**) Western blot was performed for TLR4 protein expression in fresh surgical GC specimens (T) and matched adjacent normal tissues (N). The statistical plots are representative of 20 independent cases (*P<0.05). (**d**) Immunohistochemical sections showed TLR4-staining patterns in normal gastric mucosa cells and GC cells (left image, × 200; right image, × 400). (**e**) Representative IHC sections of TLR4 expression in different clinical TNM stages were shown ( × 100). Histogram showed the percentage of weak, moderate and strong TLR4 staining cases in each clinical stage

We performed quantitative real-time PCR (qRT-PCR) to screen the expression levels of *TLR1-10* in paired fresh tumor tissues and normal gastric tissue samples isolated from 10 patients with GC. Among the *TLRs* tested, *TLR4* was higher in GC tissues than that in normal gastric tissues (Figure 1a), and *TLR4* was also the most abundantly expressed TLR in GC tissues (Figure 1b). To confirm TLR4 expression in GC tissue, we examined the protein expression level of TLR4 in paired GC tissues and adjacent normal tissues isolated from another set of 20 patients with GC. Immunoblot showed significantly increased TLR4 in GC tissues compared with matched adjacent normal tissues (Figure 1c). Moreover, paraffin-embedded sections of normal human gastric tissues and GC tissues were stained to determine their expression of TLR4 using immunohistochemistry (IHC). Normal gastric epithelia and stroma were generally negative for TLR4 (Figure 1d). In cancer cells, higher expression of TLR4 was localized in the cytoplasm and in the cell membrane (Figure 1e). A strong positive staining of TLR4 was characteristic for advanced-stage tumors (TNM III or IV), whereas moderate or weak staining was characteristic for early-stage tumors (TNM I or II) (Figure 1e). The clinicopathological characteristics of the patients were summarized in Supplementary Table 1, and the data showed that the expression of TLR4 correlated with the tumor stages of GC.

### TLR4 signaling activation promotes GC cell proliferation

**Figure 2:**
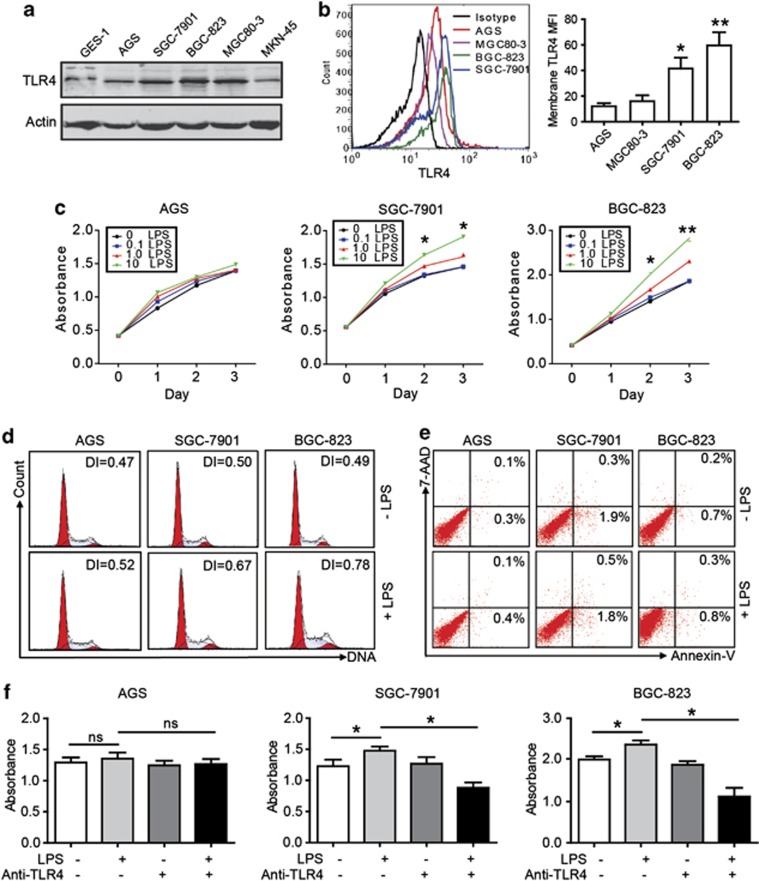
GC cell expresses TLR4 and TLR4 signaling accounts for the GC cell proliferation. (**a**) TLR4 expression levels of GC cell lines were analyzed by western blot. (**b**) TLR4 expression in GC cell membranes was evaluated by FCM. MFI, mean fluorescence intensity. Results shown are the means±S.E.M.s of three independent experiments (*P<0.05; **P<0.01, compared with AGS). (**c**) GC cells were treated with or without LPS at various concentrations (0.1–10 *μ*g/ml), and a dose-dependent effect of LPS on GC cells proliferation was measured (*P<0.05, compared with untreated control cell). (**d**) DI showed cellular DNA content of GC cells with or without LPS treatment after 48 h by FCM. (**e**) GC cells were treated with LPS (10 *μ*g/ml) for 48 h and then labeled with annexin V and 7-AAD for apoptosis assay. (**f**) GC cells were treated with LPS (10 *μ*g/ml) and/or an anti-TLR4 blocking antibody for cell proliferation. The bars show cell counts from five independent experiments (ns, not significant; *P<0.05; **P<0.01)

To identify the expression of TLR4 in GC cell lines, we performed the immunoblot to detect the expression level of TLR4 in GC cell lines. Our results showed that the highest level of protein was found in BGC-823 cell and lower level in AGS cell (Figure 2a). As the adapter, the membrane expression of TLR4 has important role in the function assay. Using flow cytometry (FCM), we also found higher level of membrane TLR4 protein in BGC-823 and SGC-7901 cell than that in other GC cell lines (Figure 2b). To determine the function of TLR4, the effects of various concentrations of LPS (0.1–10 *μ*g/ml) on tumor cell proliferation were studied in three GC cell lines with different TLR4 expression levels. As shown in Figure 2c, LPS significantly enhanced GC cell proliferation in BGC-823 and SGC-7901 cell than that in AGS cell, which had lower TLR4 expression level. Moreover, LPS stimulated the growth of BGC-823 and SGC-7901 in a concentration-dependent manner (Figure 2c). Furthermore, we studied the cell cycle of three GC cell lines exposed to LPS stimulation. LPS drove BGC-823 and SGC-7901 cell to undergo proliferation with a greatly increased DNA index (DI), which indicates the ratio of proliferating cells to resting cells (Figure 2d). The effect of LPS on GC cell apoptosis was also analyzed, and we did not find significant change after LPS exposure (Figure 2e). To confirm the influence of TLR4 signaling on GC cell propagation, a neutralizing TLR4 Ab was used. The proliferation of the BGC-823 and SGC-7901 cell following LPS stimulation was partially abrogated in the presence of the neutralizing Ab specific for TLR4 (Figure 2f).

### LPS enhances ROS and mROS production in GC cells via the activation of TLR4 signaling

**Figure 3:**
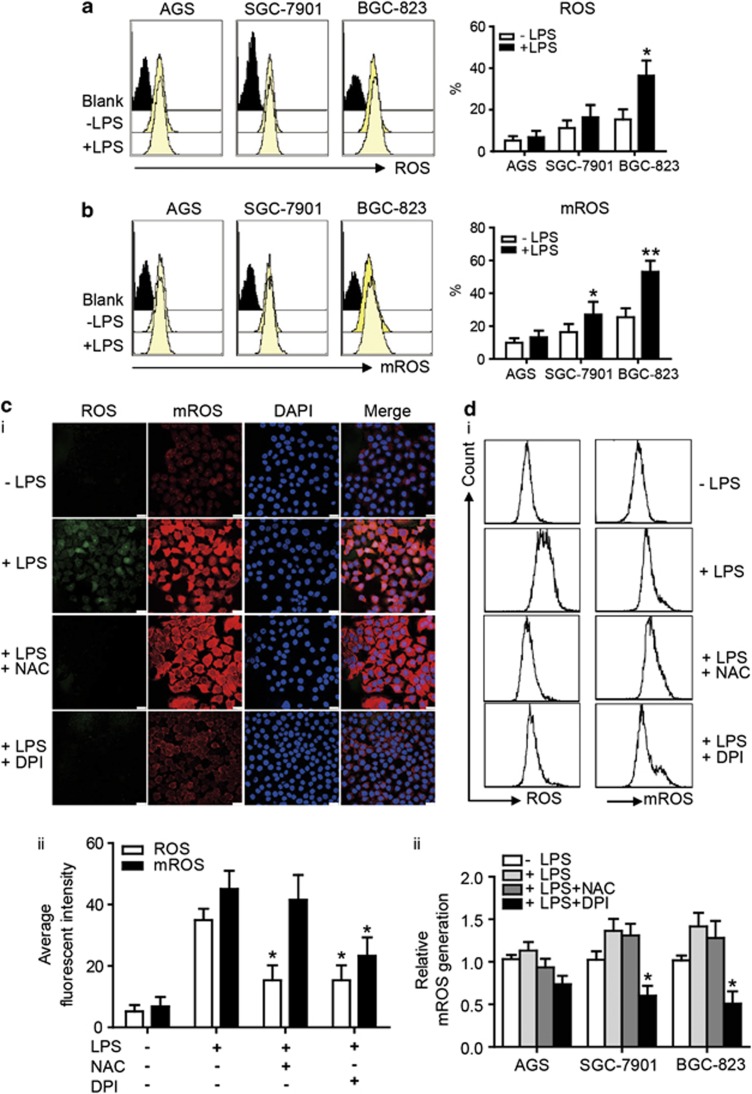
TLR4 signaling induces ROS and mROS generation in GC cell lines. (**a**) The intracellular generation of ROS in GC cells with LPS (10 *μ*g/ml) for 24 h was analyzed by CM-H2DCFDA using FCM, and the production of H2O2 was quantified (*P<0.05). (**b**) GC cells were stimulated with LPS (10 *μ*g/ml) for 24 h and labeled with MitoSOX. The quantitative analysis of mROS production by FCM was shown (*P<0.05; **P<0.01). (**c**) (i) BGC-823 cell was pretreated with NAC (10 mmol/l) or DPI (10 *μ*mol/l) for 2 h before LPS (10 *μ*g/ml) stimulation. Representative images showed ROS and mROS production by confocal microscopy. Scale bar, 20 *μ*m. (ii) The quantitative analysis showed different effects of NAC and DPI on ROS and mROS generation in BGC-823 cell (*P<0.05, compared with LPS treatment group). (**d**) (i) The generation of ROS and mROS upon LPS (10 *μ*g/ml) stimulation after NAC or DPI pretreatment was analyzed by FCM in BGC-823 cell. (ii) The quantitative analysis of mROS production was shown in AGS, SGC-7901 and BGC-823 cell (*P<0.05, compared with LPS treatment group)

ROS are essential components of the innate immune response against intracellular bacteria. To elucidate the relationship between TLR4 expression and ROS production, we measured the spontaneous cellular ROS and mROS levels using FCM, and then the change of ROS production in GC cells after LPS treatment was examined. The results showed that GC cells have spontaneous ROS generation, and LPS increased ROS production in BGC-823 and SGC-7901 cell, which have higher TLR4 levels than AGC cell (Figure 3a; Supplementary Figure 1a). To determine whether TLR signaling affected mROS production, our results showed that LPS enhanced mROS production in GC cells, especially BGC-823 (Figure 3b; Supplementary Figure 1b). To identify the source of ROS generation, we evaluated the effects of various agents (an antioxidant, *N*-Acetyl-L-cysteine (NAC), and a mitochondrial complex I and Nox inhibitor, diphenylene iodonium, DPI) on GC cells. We found that both NAC and DPI inhibited ROS generation, whereas DPI had inhibitive effects on ROS and especially mROS generation (Figures 3c–i and ii). The FCM data also confirmed these results (Figures 3d–i and ii). Taken together, these results showed the increased production of ROS and mROS in GC cells after LPS stimulation, and both the Nox system and the mitochondrial system contributed to elevated ROS generation in GC cells.

### TLR4 signaling activation promotes GC cell proliferation primarily via mROS production

**Figure 4:**
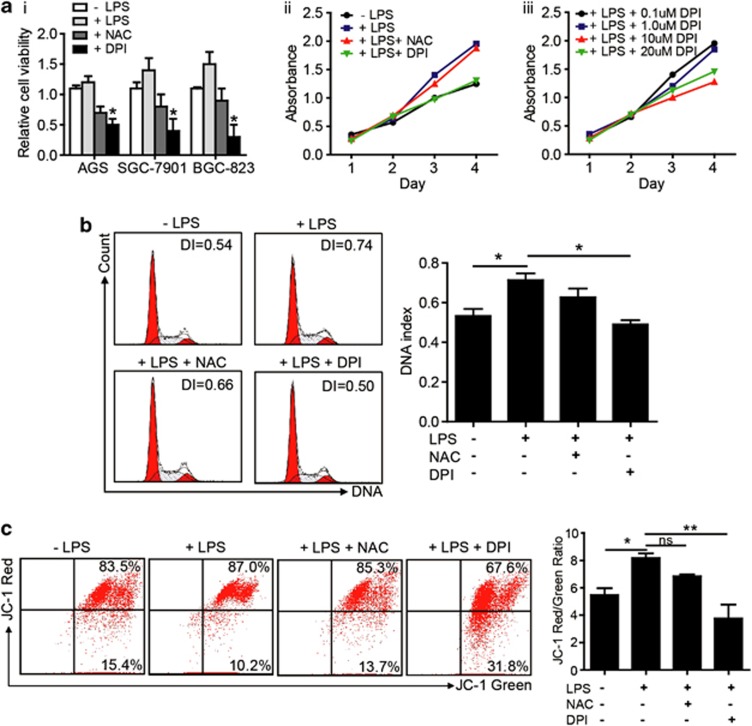
mROS generation is a critical event in TLR4 activation to promote the proliferation of GC cells. (**a**) (i) The GC cells were treated as indicated and cell viability was determined by cell counting under microscope after 3 days culture (*P<0.05, compared with LPS treatment group). (ii) BGC-823 cell was pretreated with or without NAC (10 mmol/l) or DPI (10 *μ*mol/l) for 2 h, stimulated with LPS (10 *μ*g/ml) and then cultured for 4 days. The cell proliferation rate was measured on different days. (iii) A dose-dependent intervention of DPI on BGC-823 cell proliferation was observed. (**b**) BGC-823 cell was pretreated with either NAC or DPI for 2 h, followed by LPS stimulation for 48 h. FCM was used to determine the cell cycle distribution. (**c**) BGC-823 cell was treated as indicated, labeled with JC-1 and analyzed for the mitochondrial membrane potential by FCM. The right graph showed the quantification of the JC-1 red/green ratio from five independent experiments (*P<0.05; **P<0.01)

To explore whether ROS production led to cell proliferation after LPS stimulation, we detected the GC cell growth using different ROS inhibitors. We found that DPI significantly inhibited GC cells proliferation, whereas NAC was less effective (Figures 4a–i) and DPI can reverse the proliferation of GC cell induced by LPS (Figures 4a–ii and iii). Moreover, DPI reduced mROS production and inhibited BGC-823 cell proliferation in a dose-dependent manner (Figures 4a–iii). Interestingly, agent that inhibited mROS generation was more effective in blocking cell growth compared with the ROS antioxidant agent NAC, suggesting that the inhibition of mROS generation rather than ROS neutralization might be a better strategy for intervention in the proliferation of GC cells after TLR4 signaling activation (Figure 4b). We tested whether or not mROS generator could increase the proliferation of GC cell in a dose-dependent manner, and found that excess production of mROS by rotenone did not increase the proliferation of GC cells (data not show). These results indicated that appropriate amount of mROS accounted for the GC cell proliferation with LPS stimulation. The reduction of membrane potential was accompanied by a decrease in ROS production in isolated mitochondria. To obtain better insight into the mechanism by which DPI inhibited GC cell growth through decreasing mROS, we further explored the effect of mitochondrial function on GC cell proliferation by determining the mitochondrial membrane potential using the ratio of red/green JC-1 fluorescence. Treatment with LPS resulted in a higher JC-1 fluorescence ratio, and DPI, but not NAC, reversed this effect (Figure 4c).

### mROS production is required for the regulation of NF-*κ*B p65 transcriptional activation and accounts for TLR4 signaling activation

**Figure 5:**
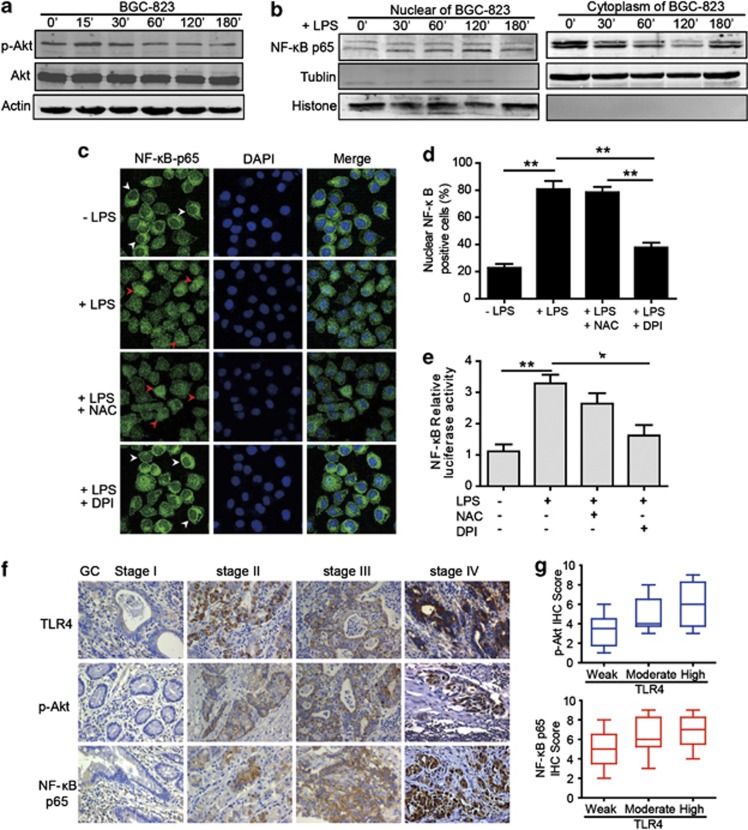
ROS production is required for LPS-induced NF-*κ*B activation. (**a**) Successive changes in Akt phosphorylation at different times after LPS treatment were shown in BGC-823 cell by western blot. (**b**) BGC-823 cell was stimulated with LPS for the indicated time, and then immunoblot for NF-*κ*B p65 was performed on the cytoplasmic and nuclear extracts. (**c**) Immunofluorescence assay of BGC-823 cell treated with LPS, NAC or DPI indicated the localization of NF-*κ*B p65 (green fluorescence, arrowheads). DAPI (blue) was used as a nuclear counter stain. White arrowheads showed that NF-*κ*B p65 mainly localized in the cytoplasm and red arrowheads indicated the nuclear translocation of NF-*κ*B p65. The original magnification was × 1000. (**d**) Graph showed the quantification of nuclear NF-*κ*B p65-positive staining. Results are presented as means±S.E.M.s (**P<0.01). (**e**) Graph showed the relative nuclear NF-*κ*B-p65 promoter activity by the dual-luciferase assay. Data are presented as means±S.E.M.s (*P<0.05; **P<0.01). (**f**) Representative IHC staining patterns of TLR4, p-Akt and NF-*κ*B p65 in GC tissues with different TNM stages were shown. The original magnification was × 400. (**g**) The box and whisker plots showed that IHC scores of p-Akt and NF-*κ*B p65 were associated with various TLR4 expression intensity (weak, *n*=6; moderate, *n*=6; and high, *n*=6)

**Figure 6:**
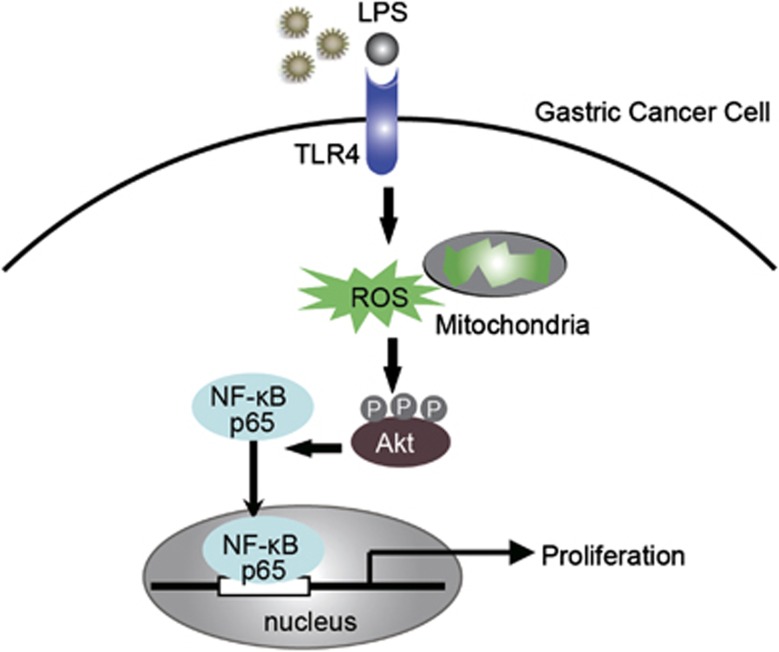
Proposed model for the role of TLR4 signaling pathway in GC cell proliferation. A drawing depicts the hypothetical role of TLR4 signaling in GC cell proliferation. We hypothesize that increased ROS generation, resulting from the activation of TLR4 signaling, mediates the activation of phosphorylated Akt, and then induces NF-*κ*B p65 nuclear translocation, which leads to GC cell proliferation

To elucidate the mechanism by which TLR4 signaling affected GC cell proliferation, we evaluated the effect of LPS on cell signaling pathway. We observed that TLR4 activation enhanced the phosphorylation of Akt in BGC-823 cell without affecting total Akt level (Figure 5a). We also found significant increase in NF-*κ*B p65 subunit translocation into the nucleus after LPS treatment (Figure 5b). The highest frequency of p65-positive cells and the highest intensity of fluorescence in the nuclei were observed in BGC-823 with TLR4 activation (Figure 5c). A variety of results now support that ROS can modulate various cellular events, from gene expression to cellular proliferation. This hypothesis was supported by our results indicating that LPS-induced NF-*κ*B p65 activation in GC cell was reversed when the cell was incubated with DPI (Figures 5c and d). Moreover, we did not observe the significant inhibition with NAC (Figure 5d). To examine the effect of mROS production on NF-*κ*B p65 transcriptional activation, the cell was transfected with a full-length human NF-*κ*B p65 promoter luciferase reporter plasmid. Treatment with DPI greatly inhibited the NF-*κ*B p65 reporter activity stimulated by LPS, whereas NAC was less effective (Figure 5e). These data suggest that ROS, mainly mROS, are required for NF-*κ*B p65 transcriptional activation after LPS stimulation. To verify the similar TLR4 signaling pathway proteins expression in clinical biopsies, the expression of TLR4, p-Akt and NF-*κ*B p65 were performed by IHC in the GC tissues. The IHC data showed that the p-Akt and NF-*κ*B p65 expression was substantially greater in advanced-stage GC tissues than in early stages, and a strongly positive NF-*κ*B p65 expression in the nucleus was observed in advanced GC (Figure 5f). With regard to TLR4 expression, p-Akt and NF-*κ*B p65 staining scores were much higher for patients with higher TLR4 expression than those with weak TLR4 expression (Figure 5g). On the basis of the above experimental and clinical results, we hypothesize that TLR4 signaling activation increases mROS generation, which leads to the phosphorylation of Akt protein and promote the activation and nuclear translocation of NF-*κ*B p65, mediating several signaling pathways that could potentially regulate various phenotypic features of GC cells (Figure 6).

## Discussion

In this study, our results suggested that TLR4 expression in GC correlated with tumor stages and activation of TLR4 contributed to GC cell proliferation via mROS production. An elevated level of spontaneous ROS generation was found in GC cells, and increased ROS production by TLR4 signaling was critical for the malignant phenotype of GC. We also found that after TLR4 activation, ROS may originate from the cytosolic NADPH oxidase and mitochondria in the GC cells and cause the NF-*κ*B p65 translocation. Moreover, DPI blocked proliferation and caused cell cycle arrest mainly by inhibiting mROS production. Collectively, we demonstrated that the engagement of TLR4 resulted in GC cell proliferation and augmented the mROS-mediated signaling pathway.

Chronic inflammation is a key contributor to carcinogenesis in various organs including the stomach, colon, lung and liver. Given the relationship between inflammation and carcinogenesis, recent studies have addressed the role of TLRs in inflammation-associated carcinogenesis in various cancers including GC.^18^ However, controversies exist concerning the role of TLRs in tumor progression. Some reports have provided evidence that TLR4 facilitates tumor progression and angiogenesis,^19, 20, 21^ whereas others suggest that TLR4 signaling inhibits tumor progression.^22, 23^ In this study, we confirmed that GC cells expressed high TLR4 level that correlated with the tumor stages. Our results described the involvement of TLR4 signaling in promoting tumor development by showing that LPS can significantly induce human GC cells to proliferate. Furthermore, LPS stimulated GC cell proliferation correlated with TLR4 expression, and neutralizing TLR4 Ab can reverse the effect of LPS stimulation.

To further elucidate the mechanism of TLR4 on GC proliferation, the role of inherent oxidative stress in GC progression has been characterized *in vitro*. There is evidence suggesting a role for oxidative stress in the pathogenesis of cancer.^24, 25^ However, the relationship between TLR signaling and ROS generation has not been elucidated in GC. In this study, we showed that ROS account for the TLR4 signaling-mediated activation of GC cell proliferation. Given that the mitochondria are a major source of ROS and altered mitochondrial bioenergetics might underlie the development of cancer, we used general antioxidants (NAC and DPI) to identify the source of ROS production. Our results showed that DPI effectively reduced mitochondrial oxygen consumption in GC cells, whereas NAC selectively affected ROS. Although the effectiveness of DPI often attributed to the inhibition of Noxs, our data and the results of others suggest that DPI is a potent inhibitor of oxygen consumption and mROS generation in a dose-dependent manner.^17, 26, 27^ The inhibition of mROS generation by DPI caused cell cycle arrest. Notably, mROS generation accounted for GC cell proliferation; however, excessive mROS did not promote GC growth and instead induced apoptosis. Surprisingly, although rotenone has been reported as an effective electron transport inhibitor, our data indicated that it did not decrease mitochondrial ROS production in GC cells. On the contrary, rotenone induced high levels of mROS production and accounted for GC cell death, which was consistent with the concept that rotenone both increases and decreases mitochondrial ROS production in a variety of cell types.^28, 29^ Altogether, these results showed that the blocking of mROS production rather than ROS neutralization resulted in cell cycle arrest and a loss of mitochondrial potential, which are plausible reasons for the decreased cell viability.

ROS can act as secondary messenger and control various signaling cascades.^14, 15, 30, 31, 32, 33^ In our studies, LPS stimulation caused the activation of phosphorylated Akt (p-Akt) and induced the nuclear translocation of NF-*κ*B p65 in GC cells. Moreover, we also found that the consistent protein expression patterns of p-Akt and NF-*κ*B p65 were highly related to the TLR4 expression in the GC tissue biopsies. These results suggest that DPI blocks the production of mROS in GC cell lines, thus inhibiting NF-*κ*B p65 translocation. Because Akt enhances cell survival by countering mitochondrial apoptotic signals, we evaluated the effect of DPI on mitochondrial membrane potential. The results of our study showed a loss of mitochondrial membrane potential in DPI-treated cells. These results offer one possible regulatory mechanism for the cell survival modulated by ROS/mROS generation. On the basis of these results and discussions, we speculate that increased mROS generation as a result of the activation of TLR4 signaling mediates several signaling pathways that could potentially regulate various phenotypic features of GC cell. The proposed mechanism for the progression of GC induced by TLR4 expression in GC cells is as follows: activated TLR4 signaling induces the formation of more ROS, especially mROS, and the resulting oxidative stress contributes to the upregulation of phosphorylated Akt, NF-*κ*B p65 activation and nuclear translocation, which leads to GC cell proliferation. However, future research should more specifically address the mechanism by which TLR4 signaling activation enhances mitochondrial respiration.

In summary, these data provide conclusive evidence that TLR4 signaling exerts a profound influence on GC progression. These results provide a new mechanism for mROS production in GC cells after TLR4 signaling and show direct role for mROS in regulating tumor growth, which indicate that TLR4 may regulate tumor growth via mROS production and the induction of signaling cascades. Moreover, our study provides a strong rationale for targeting TLR4 signaling or mROS production for the prevention of GC.

## Materials and Methods

### Patients and tissue samples

Gastric tumors were obtained from a cohort of patients treated at Xinhua Hospital, affiliated with Shanghai Jiaotong University School of Medicine, China, between 2005 and 2011. The median age of the patients was 57.6 years. All patients were diagnosed by pathological analyses based on the International Union Against Cancer defined TNM criteria. The study protocol conformed to the ethical guidelines of the Declaration of Helsinki and was approved by the Institutional Review Board and Ethics Committee of Xinhua Hospital, Shanghai Jiaotong University School of Medicine. Before inclusion in the study, all patients provided written informed consent. The clinicopathologic characteristics of the patients are summarized in Supplementary Table 1.

### Cell culture and treatment conditions

Human GC cell lines MGC80-3, SGC-7901, BGC-823 and AGS were obtained from the Chinese Academy of Sciences Cell Bank of Type Culture Collection. Human GC cell line MKN-45 and non-cancerous gastric epithelial cell line GES-1 were provided by Beijing Institute for Cancer Research. The cells were routinely cultured in DMEM media supplemented with 10% fetal calf serum, 100 U/ml penicillin and 100 *μ*g/ml streptomycin (Gibco, Grand Island, NY, USA) in 5% CO_2_ at 37 °C. The cells used for our experiments were in the log-phase of growth and were negative for mycoplasma and endotoxin, as confirmed by PCR (Mycoplasma Tissue Culture Detection kit, Gen-Probe, San Diego, CA, USA) and the Limulus Amebocyte Lysate assay (Cambrex, Walkersville, MD, USA), respectively. LPS (ALX-581-010-L002, Enzo Life science, Farmingdale, NY, USA) was added to the tumor cells at concentrations of 0.1, 1.0 and 10 *μ*g/ml. NAC was used as an antioxidant and DPI was used as a mitochondrial complex I and Nox inhibitor. NAC (10 mmol/l) or DPI (10 *μ*mol/l) was applied to the GC cells at least 2 h before LPS stimulation. In blocking experiment, anti-TLR4 Ab (IMG-417A, IMGENEX, San Diego, CA, USA) was used as neutralization antibody.

### Quantitative RT-PCR

Quantitative real-time PCR analysis was carried out to detect the mRNA expression of *TLR1-10*. Total RNA extraction from GC tissue was performed with Trizol Reagent (Invitrogen, Grand Island, NY, USA). Then, RNA was reverse transcribed and was quantified by real-time PCR using the Applied Biosystems, Foster City, CA, 7500 System (Applied Biosystems, Foster City, CA, USA). All protocols were used as we have described previously.^34^ Primers sequences of *TLR1-10* are described in Supplementary Table 2.

### Immunohistochemical staining

Standard immunohistochemical procedures were performed using the VECTASTAIN Elite ABC system (Vector Laboratories, Burlingame, CA, USA) according to the manufacturer's protocol. Anti-TLR4 polyclonal antibody (Abcam, Cambridge, MA, USA), anti-phosphorylated Akt (Cell Signaling Technology, Beverly, MA, USA) and anti-NF-*κ*B p65 (Santa Cruz Biotechnology Inc., Santa Cruz, CA, USA) were used as primary antibodies. The staining intensity (0, no staining; 1, weak staining; 2, moderate staining; and 3, intense staining) and the proportion of stained cells (0, no staining; 1, <10% staining; 2, between 11 and 33% staining; 3, between 34 and 66% staining; and 4, >67% staining) were semiquantitatively determined. The intensity and the percentage of positive cell scores were multiplied (0–12) and classified into three groups: weak (0–4), moderate (5–8) and strong (9–12). All slides were scored by two observers blinded to the pathology and the clinical features. In cases where the score difference was equal to or exceeding 2, the slides were re-examined and a consensus was reached by the observers.

### Western blot analysis

Western blot analyses were performed as previously described.^35^ Briefly, the cells were lysed in equal volumes of ice-cold lysis buffer and a protease inhibitor cocktail. Nuclear extracts and cytoplasmic extracts were prepared using NE-PER nuclear and cytoplasmic extraction Reagents (Thermo Scientific, Rockford, IL, USA) when needed. Cell homogenates were boiled and the proteins were separated by SDS-PAGE. After overnight incubation at 4 °C with anti-phosphorylated Akt (p-Akt) (Clone: 193H12), anti-Akt (Clone: 067E7), anti-NF-*κ*B p65 (Clone:93H1) (Cell Signaling Technology, Beverly, MA, USA) or anti-TLR4 antibody (Abcam), the membranes were incubated with IRDye 800 goat anti-rabbit or IRDye 680 goat anti-mouse secondary antibodies (LI-COR Biosciences, Lincoln, NE, USA). The targeted proteins were detected and quantified on a Li-COR Odyssey infrared imaging system (LI-COR Biosciences).

### Cell proliferation

Tumor cells plated overnight in 96-well plates at a density of 3 × 10^3^ per well were incubated with fresh medium or a medium supplemented with LPS at various working concentrations with or without NAC and DPI. Cell viability was determined using the CCK-8 Cell Proliferation Assay (Dojindo, Tokyo, Japan) according to the manufacturer's instructions. In some experiment, the viability and the numbers of tumor cells were determined using microscope counts in the presence of a trypan blue dye using tumor cells harvested after treatment with TripLE Select solution (Invitrogen) on day 3 of culture.

### Determination of ROS and mROS superoxide levels

To visualize total intracellular levels of ROS and the mROS superoxide, immunofluorescence assay and FCM analysis were performed. Log-phase cells were grown on 24-well plates and treated with various agonists or stimulated with LPS as indicated. The culture medium was removed and the cells were washed with PBS and incubated with CM-H2DCFDA (to measure the total cellular H_2_O_2_ levels) (Invitrogen) at a final concentration of 2.5 mM and/or MitoSOX (to measure the mROS superoxide levels) (Invitrogen) at a final concentration of 5 *μ*M in serum-free DMEM for 30 min at 37 °C. For immunofluorescence assay, the cells were mounted with ProLong Gold Antifade Reagent with DAPI (Invitrogen) and images were acquired using a Leika SP5 confocal microscope (Leika Systems, Mannheim, Germany). For FCM analysis, the cells were digested and subjected to FACS Canto II cytometer (BD Biosciences, San Jose, CA, USA). To control for baseline dye fluorescence, samples from each experiment were left unstimulated but stained according to the above procedure. All experiments shown are representative of three independent experiments.

### Measurement of mitochondrial transmembrane potential

The change in mitochondrial transmembrane potential induced by DPI and NAC in GC cell lines was observed with JC-1 fluorescent probes (Invitrogen) by FCM. Cells (1 × 10^4^) plated in 24-well plates were treated with either DPI or NAC for 8 h, labeled with JC-1 (2.5 *μ*g/ml) for 15 min at 37 °C, washed with PBS and analyzed on a FCM using 488 nm excitation with 530 and 585 nm band pass emission filters. The changes in color from red to green were quantified and analyzed.

### Cell cycle distribution and DNA index determination

The DNA content and the cell cycle phase distribution were assessed by FCM. GC cells were exposed to LPS, DPI or NAC for 8 h. The cells were washed in PBS and stained with DNA Prep Reagents kit (Beckman Coulter, Fullerton, CA, USA). After the exclusion of dead cells by light scattering measurements, 10 000 cells were analyzed for fluorescence intensity with FACS Canto II cytometer (BD Biosciences). The data were analyzed using the ModFit LT software (Verify Software House, Topsham, ME, USA), and the DI was defined as the ratio of the G2/M and S phase cells to the G0/G1 phase cells.

### Apoptosis assay

Apoptosis was monitored by annexin V and 7-AAD (BD Biosciences) staining according to the manufacturer's instructions. The cells were treated with or without LPS and either DPI or NAC for 8 h then labeled with annexin V and 7-AAD. Apoptotic cells were defined as the population that was positive for annexin V and negative for 7-AAD. FCM experiments were conducted by FACS Canto II cytometer (BD Biosciences).

### Immunofluorescence assay

Cells were plated on the cover slips and cultured at up to 50–60% confluence, and then cells were washed with PBS and fixed with fresh 4% paraformaldehyde solution for 15 min at room temperature. Cells were then washed twice with PBS, followed by incubation in 10% normal rabbit serum blocking solution for 20 min at room temperature in a humidified chamber. Cells were incubated in the specific primary antibodies against NF-*κ*B p65 primary antibody diluted in PBS (1 : 400) for 2 h at room temperature in a humidified chamber. Cells were washed three times in PBS and incubated in Alexa Fluor 488-conjugated goat anti-rabbit IgG (Invitrogen) for 45 min at room temperature in a humidified chamber. The cells were then washed in PBS, mounted with ProLong Gold Antifade Reagent with DAPI (Invitrogen). Images were acquired using a Leika SP5 confocal microscope (Leika Systems) with 10 fields of view. All images were analyzed by Image J software (US National Institutes of Health, Bethesda, MD, USA) previously described.^36^

### Luciferase assay

BGC-823 cell was plated in 24-well plates and transfected with the NF-*κ*B-responsive luciferase reporter construct (NF-*κ*B p65-Luc) and its control plasmid (pRL-TK). At 24 h post transfection, cells were incubated with 10 *μ*g/ml of LPS for 24 h and luciferase activities were measured using a Dual-Luciferase Reporter Assay System (Promega, Madison, WI, USA) and a microplate luminometer (Promega). The firefly luciferase activities were corrected by the corresponding renilla luciferase activities. Results are representative of three independent experiments.

### Statistical analysis

The data were expressed as the mean±S.E. of means (S.E.M.). The statistical significance of the difference between two means was assessed using Student's *t*-test, and the one-way ANOVA with Tukey's post test was performed for multiple comparisons. All the statistical analyses were performed using GraphPad Prism version 5.0 for Windows (GraphPad Software, San Diego, CA, USA), and statistical significance was set at *P<0.05; **P<0.01.

## Abbreviations

TLRs: Toll-like receptors
TLR4: Toll-like receptor 4
LPS: lipopolysaccharide
ROS: reactive oxygen species
mROS: mitochondrial reactive oxygen species
NAC: *N*-Acetyl-L-cysteine
DPI: diphenylene iodonium
GC: gastric cancer
NF-*κ*B: nuclear factor-*κ*B
qRT-PCR: quantitative real-time PCR
FCM: flow cytometry
MFI: mean fluorescence intensity
IHC: immunohistochemistry
DI: DNA index
