# Supplementary material for: Activation of TLR4 signaling promotes gastric cancer progression by inducing mitochondrial ROS production
Source: Cell Death Dis. 2013 Sep 12;4(9):e794–. doi: 10.1038/cddis.2013.334 (PMC3789192; doi:10.1038/cddis.2013.334)
Supplement: Supplementary Information [file cddis2013334x1.doc]

**Supplementary Information**

**Activation of TLR4 signaling promotes gastric cancer progression by enhancing mitochondrial ROS production**

Xiangliang Yuan^1, 4^, Yunlan Zhou^1, 4^, Weiwei Wang^1^, Junhunoa Li^1^, Guohua Xie^1^, Yan Zhao^1^, Dakang Xu^2, 3^, *, and Lisong Shen^1^,*

**Inventory of Supplementary Material**

**1) Supplementary Table 1**

**1) Supplementary Table 2**

**2) Supplementary Figure 1 (related to Figure 3)**

**Supplementary Table 1. TLR4 expression and clinicopathological findings in gastric cancer**

| **Characteristics** | **N（%）** | **TLR4 Expression** | | | ***P* value** |
| --- | --- | --- | --- | --- | --- |
|  |  | **Weak** | **Moderate** | **Strong** |  |
| Age |  |  |  |  | > 0.05 |
| <50 | 25 (44.6%) | 8 | 10 | 7 |  |
| ≥50 | 31 (55.4%) | 2 | 15 | 14 |  |
| Gender |  |  |  |  | > 0.05 |
| Male | 38 (67.9%) | 4 | 18 | 16 |  |
| Female | 18 (32.1%) | 6 | 7 | 5 |  |
| Differentiation |  |  |  |  | > 0.05 |
| Well | 8 (14.3%) | 5 | 3 | 0 |  |
| Medium | 12 (21.4%) | 2 | 8 | 2 |  |
| Poor | 36 (64.3%) | 3 | 14 | 19 |  |
| TNM Stage |  |  |  |  | < 0.05 |
| I  II  III  IV | 9 (16.1%)  13 (23.2%)  22 (39.3%)  12 (21.4%) | 6  4  0  0 | 3  8  12  2 | 0  1  10  10 |  |
| II | 13 (23.2%) | 4 | 8 | 1 |  |
| III | 22 (39.3%) | 0 | 12 | 10 |  |
| IV | 12 (21.4%) | 0 | 2 | 10 |  |
| Lymph node metastasis | |  |  |  | < 0.05 |
| Negative | 18 (32.1%) | 10 | 5 | 3 |  |
| Positive | 38 (67.9%) | 0 | 20 | 18 |  |

**Supplementary Table 2. Sequences of TLRs primers used for quantitative real-time PCR**

| TLRs | Primers | |
| --- | --- | --- |
| *TLR1* | Forward | CTTATAAGTGTGACTACCCGG |
|  | Reverse | CCACAATGCTCTTGCCAGG |
| *TLR2* | Forward | GTTAACAATCCGGAGGCTGC |
|  | Reverse | TTGGGAATGCAGCCTGTTAC |
| *TLR3* | Forward | GTGCCAGAAACTTCCCATGT |
|  | Reverse | CTTCCAATTGCGTGAAAACA |
| *TLR4* | Forward | CTGCAATGGATCAAGGACCA |
|  | Reverse | TCCCACTCCAGGTAAGTGTT |
| *TLR5* | Forward | TGGGGGAACTTTACAGTTCG |
|  | Reverse | CTGGGATTCTCTGAAGGGG |
| *TLR6* | Forward | GGGTTGAGAGTATAGTGGTG |
|  | Reverse | GTAGATGCAGAGGGAGGTC |
| *TLR7* | Forward | CCTCAGCCACAACCAACTG |
|  | Reverse | TTGTGTGCTCCTGGCCCC |
| *TLR8* | Forward | AAACTTGAGCCACAACAACATTT |
|  | Reverse | ATCTCCAATGTCACAGGTGC |
| *TLR9* | Forward | AACTGGCTGTTCCTGAAGTC |
|  | Reverse | TGCCGTCCATGAATAGGAAG |
| *TLR10* | Forward | AAAACTCTAAATGCGGGAAGAAA |
|  | Reverse | GAAATAAATGCGTGGAATCGGA |


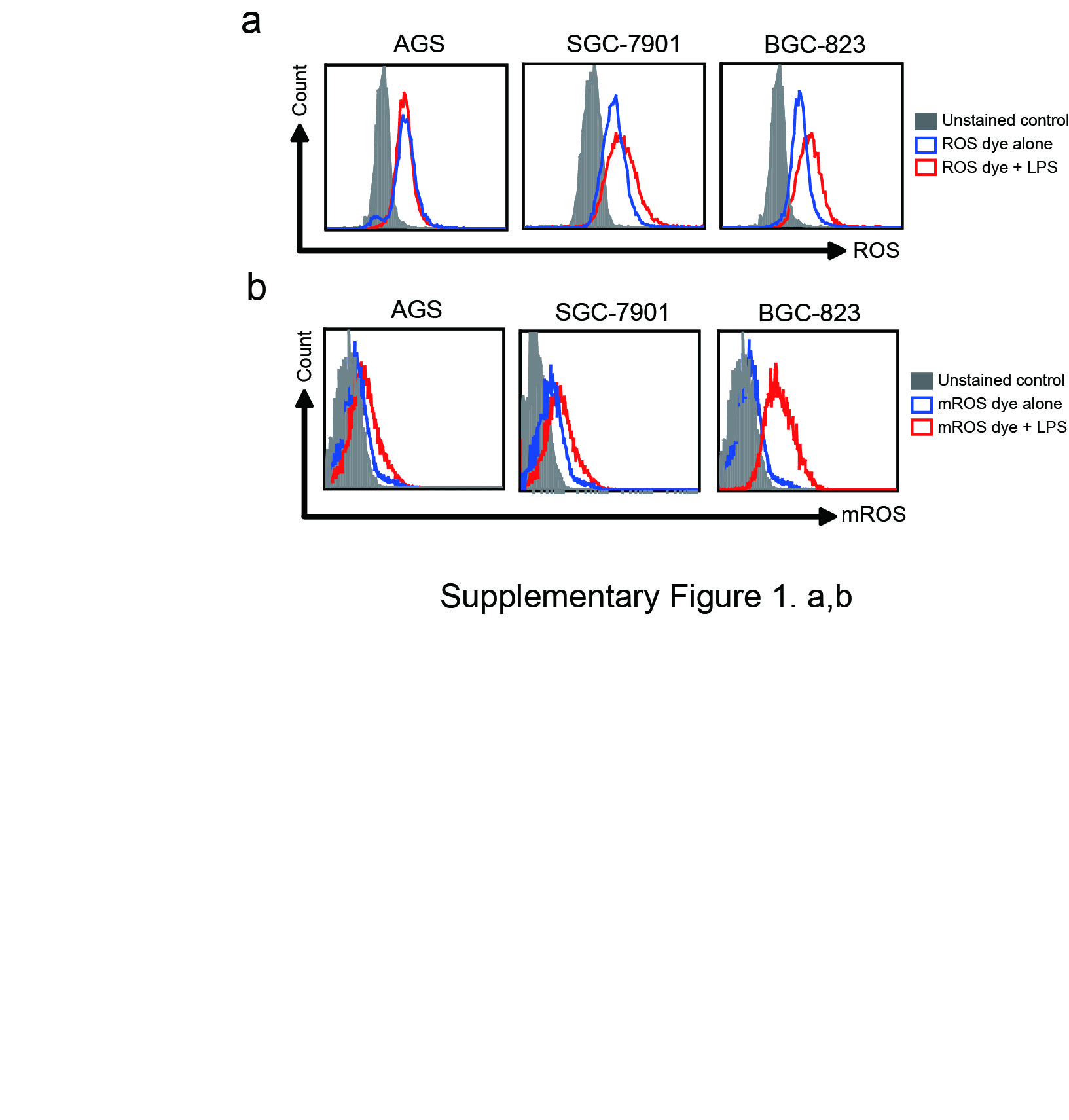


**Supplementary Figure 1. Gastric cancer cell generate endogenous ROS/mROS and TLR4 signaling induces increased ROS and mROS in gastric cancer cell. (a)** AGC, SGC-7901 and BGC-823 cell with or without LPS stimulation were labeled with CM-H2DCFDA, and intracellular generation of H_2_O_2_ was analyzed by FCM. **(b)** AGC, SGC-7901 and BGC-823 cell with or without LPS stimulation were stained with MitoSOX and analyzed the mROS level by FCM.
